# Supplementary material for: Heterogeneous impacts for malaria control from larviciding across villages and considerations for monitoring and evaluation
Source: PLoS Pathog. 2025 Jul 28;21(7):e1013287. doi: 10.1371/journal.ppat.1013287 (PMC12321148; doi:10.1371/journal.ppat.1013287)
Supplement: S1 Text — Table A. Summary of the statistical models fitted to the Kenyan entomological data. Table B. Summary of the statistical models fitted to the Côte d’Ivoire entomological data. Table C. Summary outputs from transmission model simulations using reductions in larval mosquito density as estimated empirically at the village level in Côte d’Ivoire, uncertainty in parentheses show 90% uncertainty interval. Fig A. Theoretical impact of larval source strategies that suppress Anopheles mosquitoes. Fig B. The observed data for six sentinel villages tracked during a larviciding randomized control trial in Kenya [34]. Fig C. Net use measured in the tracked cohort of children of 6-months to 10-years of age at cross sectional surveys throughout the Kenyan trial. Fig D. Summary results for the crude difference-in-difference estimates. Fig E. Larval densities analysis and model simulated results for the Côte d’Ivoire trial. (DOCX) [file ppat.1013287.s002.docx]

Heterogeneous impacts from larviciding across villages and considerations for monitoring and evaluation

Ellie Sherrard-Smith, Ulrike Fillinger, [Jean-Philippe B. Tia](mailto:bleu.tia@csrs.ci), Peter Winskill, [Benjamin G. Koudou](https://malariajournal.biomedcentral.com/articles/10.1186/s12936-024-04953-8#auth-Benjamin_G_-Koudou-Aff1-Aff2), Emile S. F. Tchicaya, Antoine Sanou, Fredros Okumu, Mercy Opiyo, Silas Majambere, Arran Hamlet, Giovanni Charles, Ben Lambert, Thomas S Churcher

S1 Text:

**Table A:** Summary of the statistical models fitted to the Kenyan entomological data. In each case, a Bayesian framework is applied that includes 4 chains with 1,000 iterations burn-in. In all models, notation $x_{1}$ is an indicator for pre (0) or post (1) intervention described by coefficient $\beta_{1}$; $x_{2}$ is an indicator for control (0) or treatment (1) arms ($\beta_{2}$); $x_{3}$ indicates species (*An. funestus* or *An. gambiae* for Kenya, $\beta_{3}$). Diagnostics shown include: i) Monte Carlo standard error (mcse) – confirming this is low relative to the posterior standard deviation otherwise uncertainty could be masked; ii) The effective posterior sample size (ESS), given MCMC draws are not independent, may show some autocorrelation. The information about the posterior are more independent when the ESS is lower and the approximate number of draws with equivalent estimated accuracy is indicated by the *n_eff_* – values above 1,000 is more than sufficient; iii) the potential scale reductor factor (Rˆ) to check within and between chain variance is similar, convergence returns a value close to 1; iv) visual checks to confirm chain convergence, following (1). Adjusted scale for priors are noted.

| Model 1: Kenya | $\theta_{i}=e^{\beta_{0}+\beta_{1}x_{1i}+{\beta_{2}x}_{2i}+ {\beta_{3}x}_{3i}+ \beta_{4}x_{1i}x_{2i}+\beta_{5}x_{1i}x_{3i}+\beta_{6}x_{2i}x_{3i} +\beta_{7}x_{1i}x_{2i}x_{3i}+(1\vert village)}$  $Y_{i}\sim NB(\mu_{i}\theta_{i}, \varphi)$  Normally distributed priors, *N ~* (*0, scale*) | | | | |
| --- | --- | --- | --- | --- | --- |
| Diagnostics: | mcse | *n_eff_* | Rˆ | Adj-scale | Visual confirmation of chain conversion |
| Intercept ($\beta_{0}$) | 0.0 | 1722 | 1.0 | | 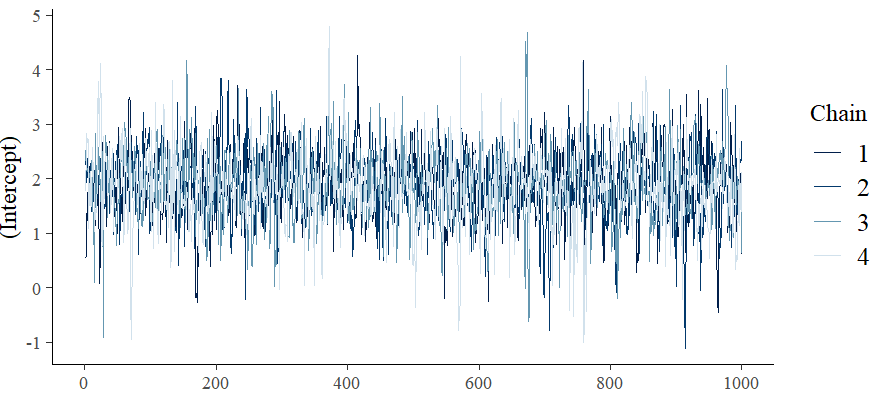 |
| $\beta_{1}$ | 0.0 | 2414 | 1.0 | 5.10 | 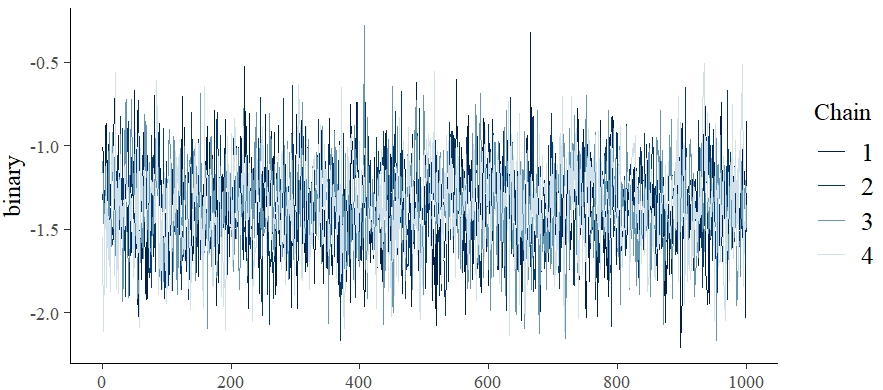 |
| $\beta_{2}$ | 0.0 | 1623 | 1.0 | 4.99 | 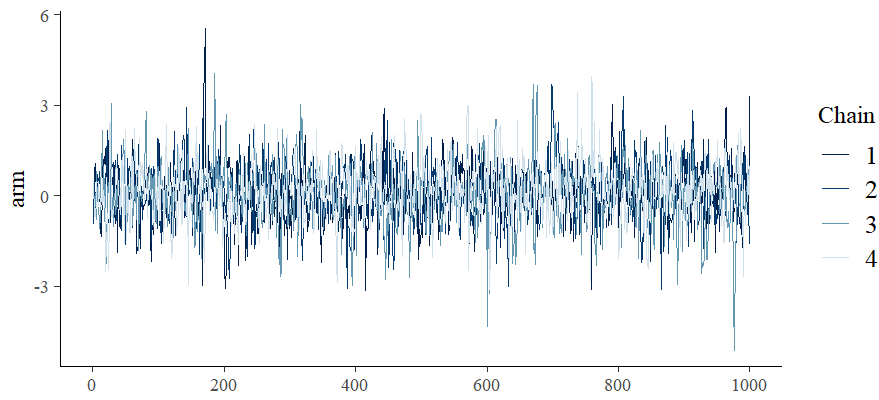 |
| $\beta_{3}$ | 0.0 | 2384 | 1.0 | 4.99 | 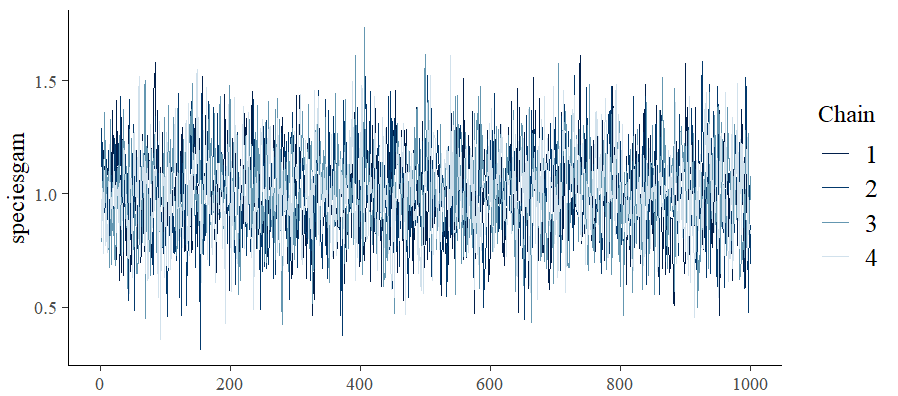 |
| $\beta_{4}$ | 0.0 | 2281 | 1.0 | 6.24 | 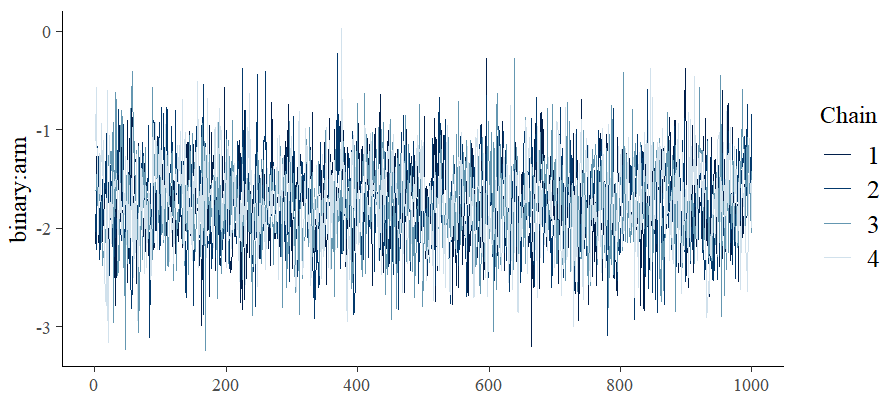 |
| $\beta_{5}$ | 0.0 | 2068 | 1.0 | 6.24 | 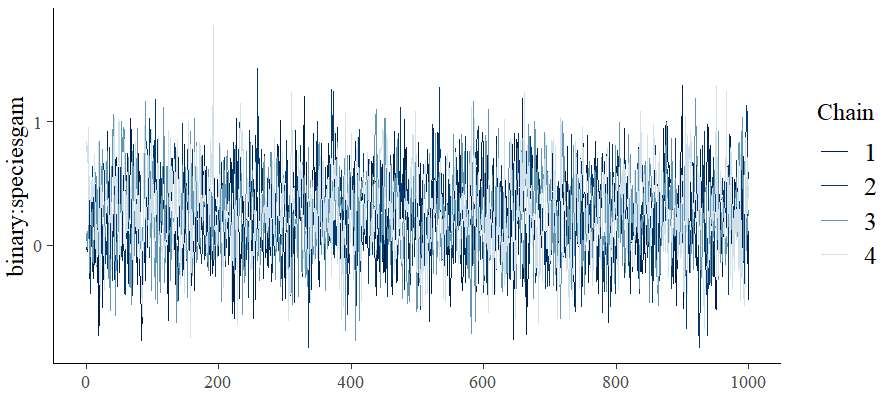 |
| $\beta_{6}$ | 0.0 | 2479 | 1.0 | 5.77 | 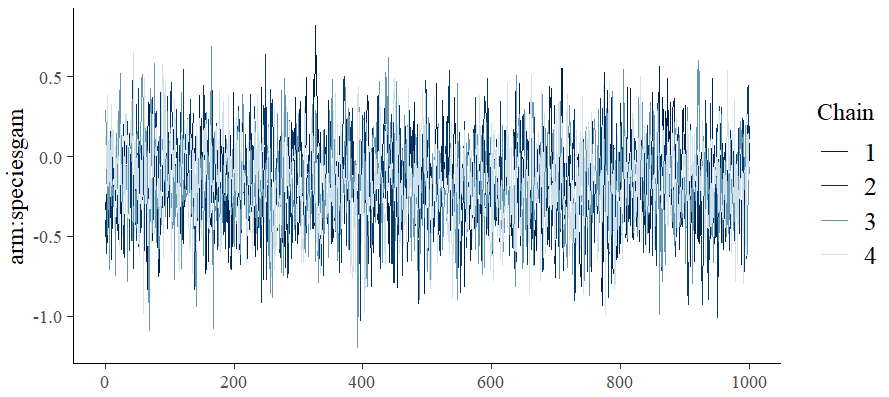 |
| $\beta_{7}$ | 0.0 | 2099 | 1.0 | 8.32 | 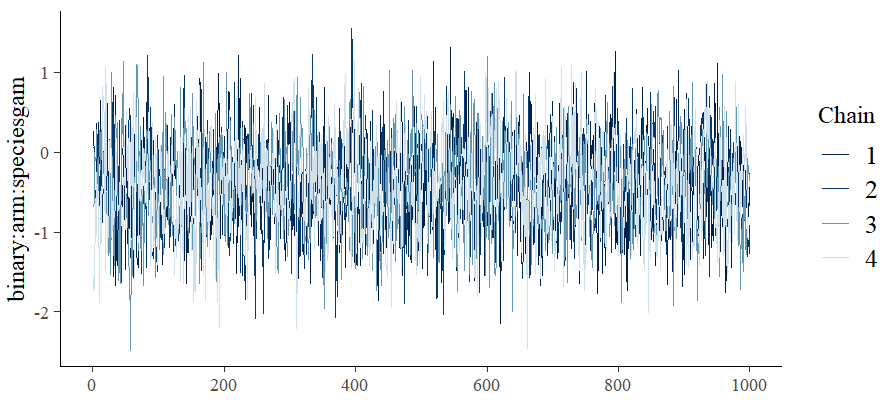 |
| b[(intercept) Musilongo]  b[(intercept) Kimingini]  b[(intercept) Kezege]  b[(intercept) Wamondo]  b[(intercept) Emutete]  b[(intercept) Wakikuyu] | 0.0 | 1875  1771  1853  1827  1839  1816 | 1.0 | | 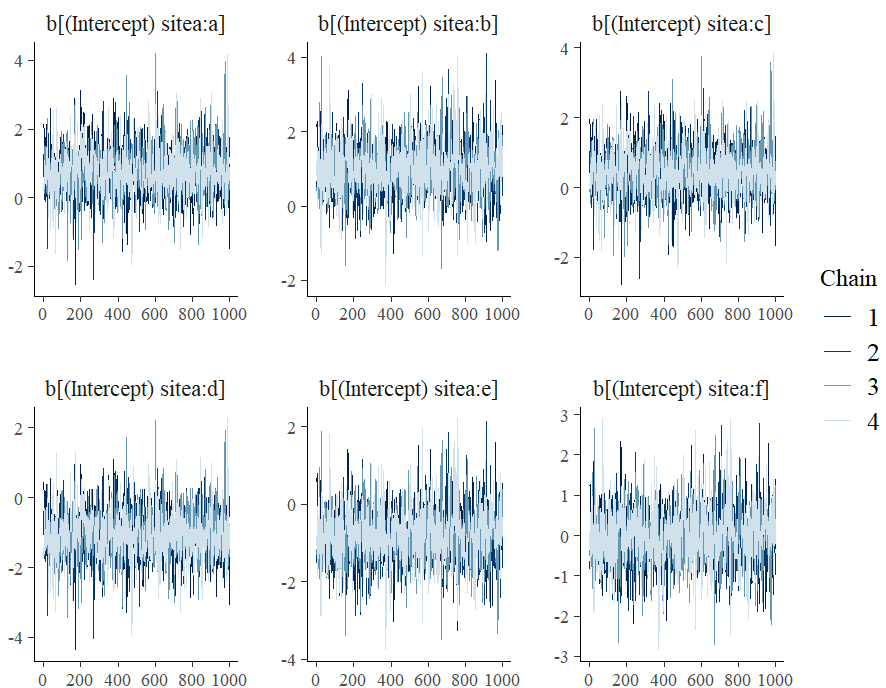 |
| Reciprocal dispersion  Sigma[Musilongo:(intercept), (intercept)] | 0.0  0.0 | 4696  1449 | 1.0  1.0 | | 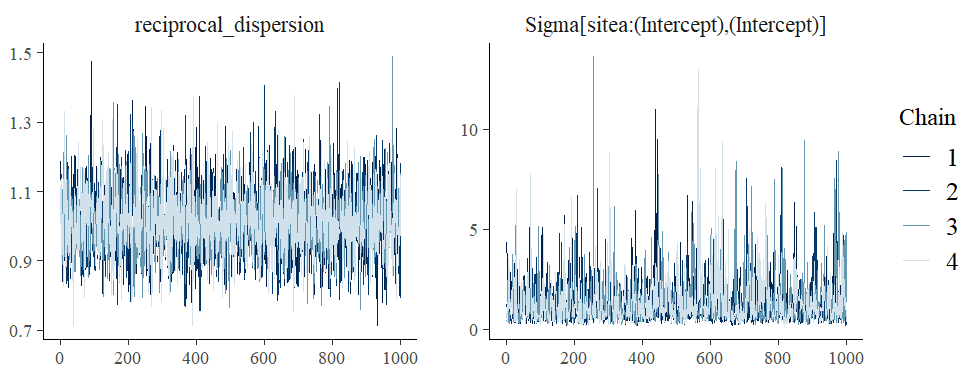 |
| 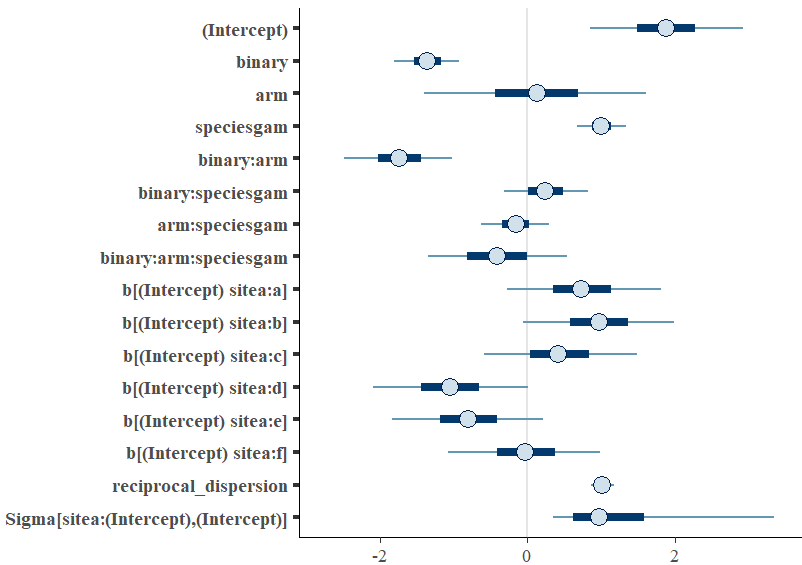 | | | | | |
| Model 2: Kenya | $\theta_{i}=e^{\beta_{0}+\beta_{1}x_{1i}+{\beta_{2}x}_{2i}+ {\beta_{3}x}_{3i}+ \beta_{4}x_{1i}x_{2i}+\beta_{5}x_{1i}x_{3i}+\beta_{6}x_{2i}x_{3i} +\beta_{7}x_{1i}x_{2i}x_{3i}}$  $Y_{i}\sim NB(\mu_{i}\theta_{i}, \varphi)$  Normally distributed priors, *N ~* (*0, scale*) | | | | |
| Diagnostics: | mcse | *n_eff_* | Rˆ | Adj-scale | Visual confirmation of chain conversion |
| Intercept ($\beta_{0}$) | 0.0 | 2484 | 1.0 | | 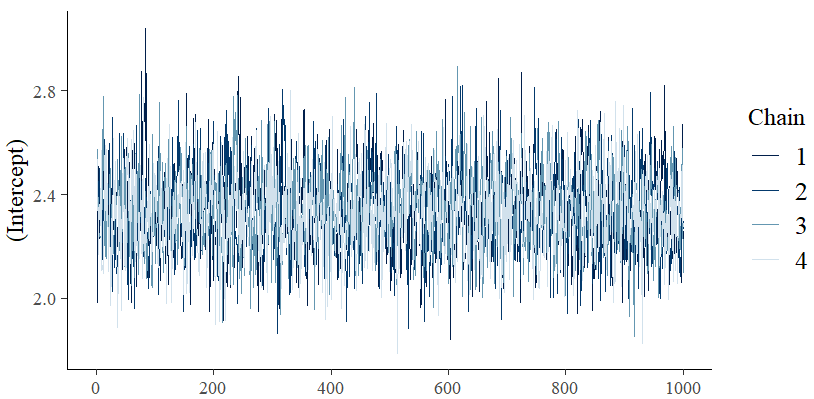 |
| $\beta_{1}$ | 0.0 | 2280 | 1.0 | 5.10 | 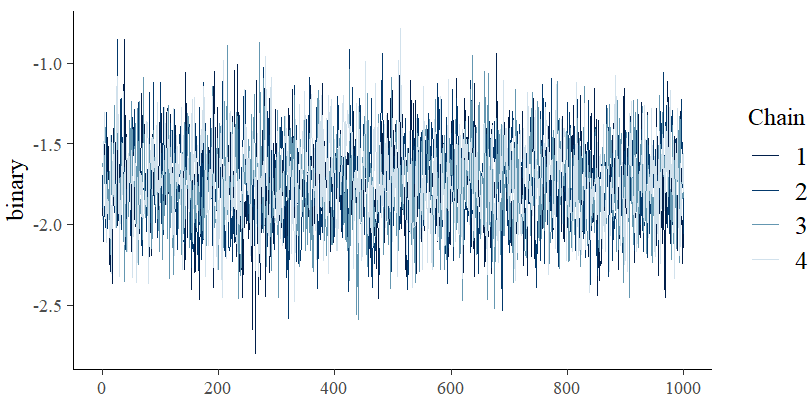 |
| $\beta_{2}$: Musilongo  $\beta_{2}$: Kezege  $\beta_{2}$: Wamondo | 0.0 | 2723  2599  2700 | 1.0  1.0  1.0 | 6.70  6.70  6.70 | 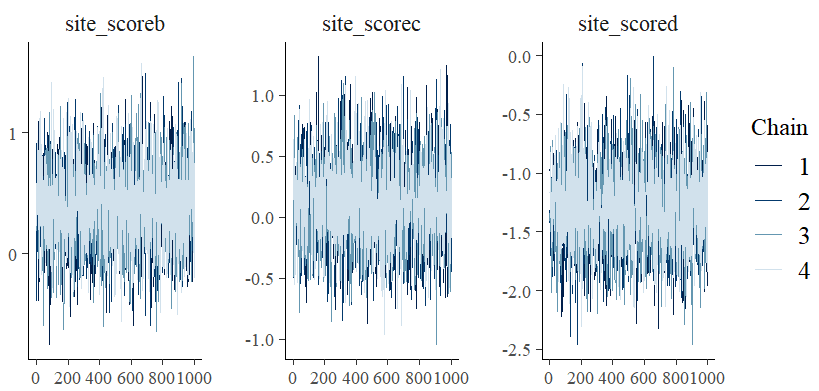 |
| $\beta_{3}$ | 0.0 | 2383 | 1.0 | 4.99 | 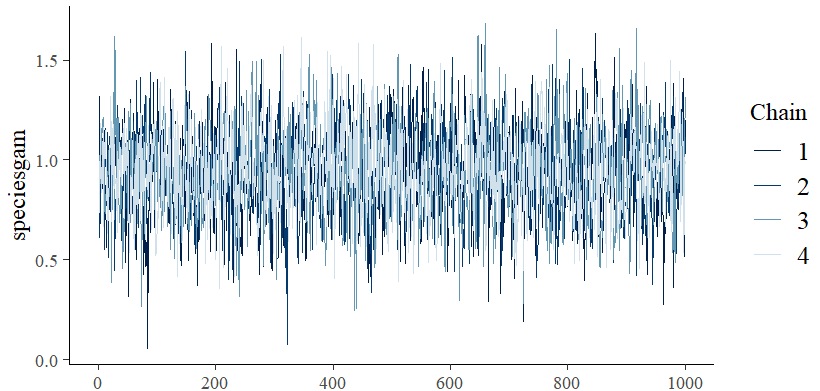 |
| $\beta_{4}$: Musilongo  $\beta_{4}$: Kezege  $\beta_{4}$: Wamondo | 0.0  0.0  0.0 | 2684  2440  1807 | 1.0  1.0  1.0 | 10.01  10.01  10.01 | 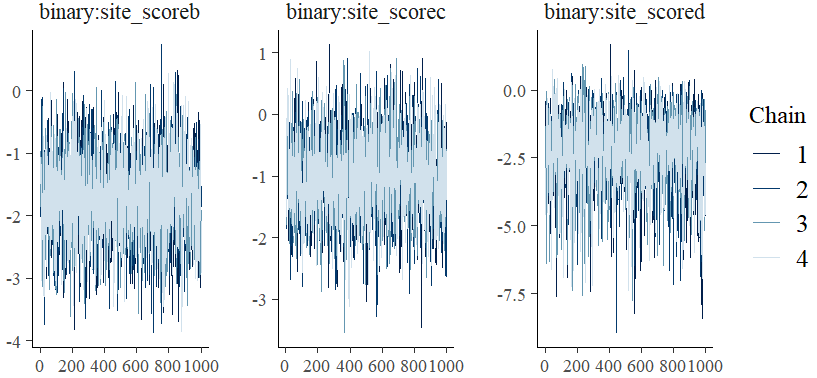 |
| $\beta_{5}$ | 0.0 | 2313 | 1.0 | 6.24 | 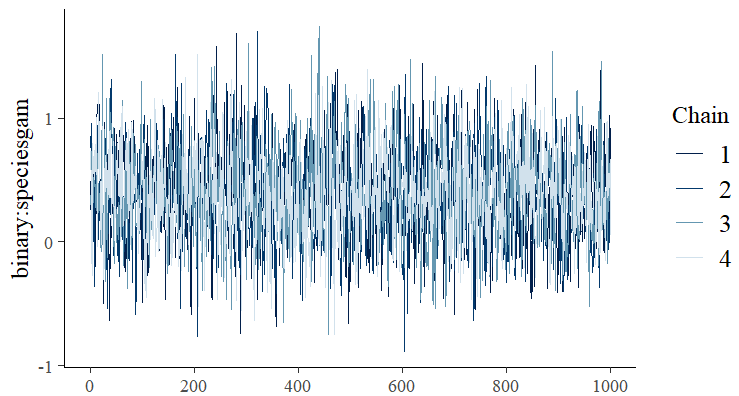 |
| $\beta_{6}$: Musilongo:gam  $\beta_{6}$: Kezege:gam  $\beta_{6}$: Wamondo:gam | 0.0  0.0  0.0 | 2471  2563  2900 | 1.0  1.0  1.0 | 9.03  9.03  9.03 | 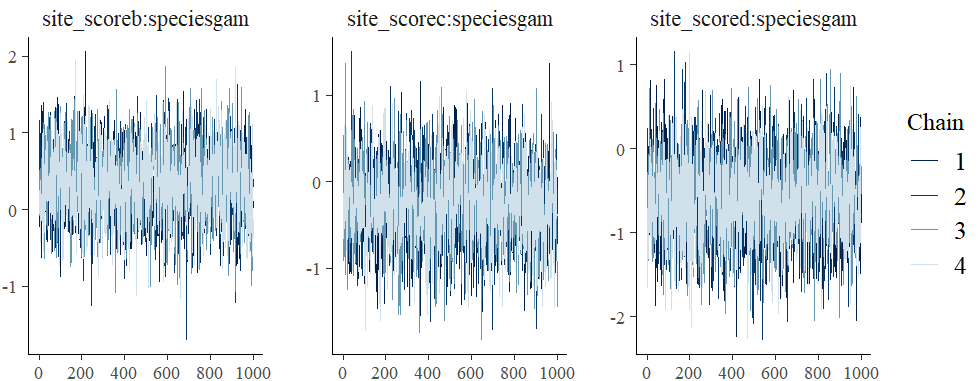 |
| $\beta_{7}$: post:Musilongo:gam  $\beta_{7}$: post:Kezege:gam  $\beta_{7}$: post:Wamondo:gam | 0.0  0.0  0.0 | 2582  2531  1926 | 1.0  1.0  1.0 | 13.91  13.91  13.91 | 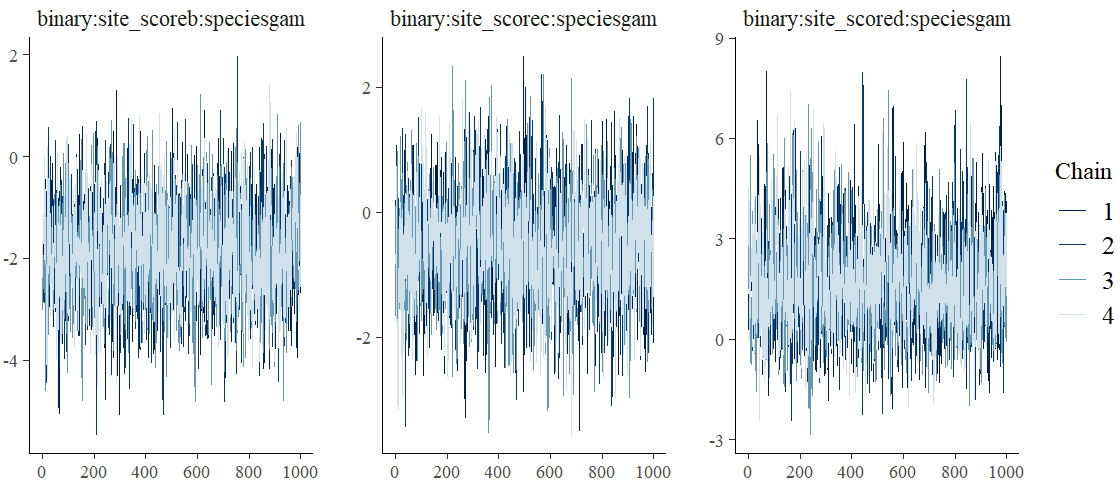 |
| Reciprocal dispersion | 0.0 | 4934 | 1.0 | | 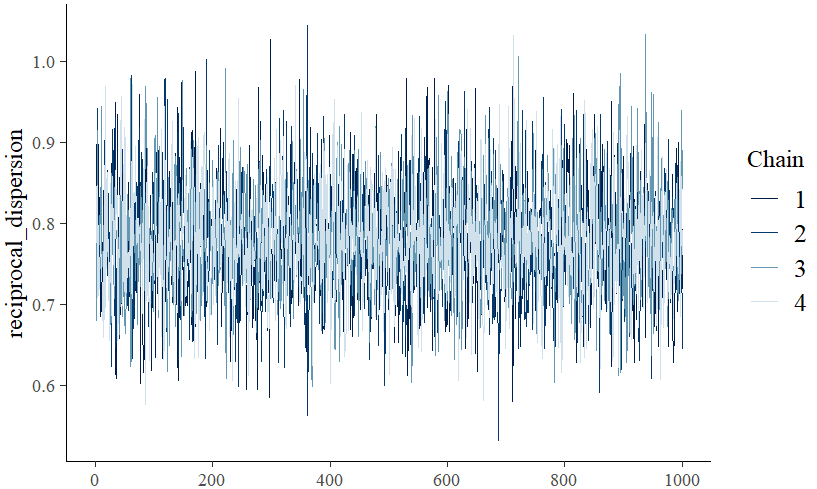 |
| 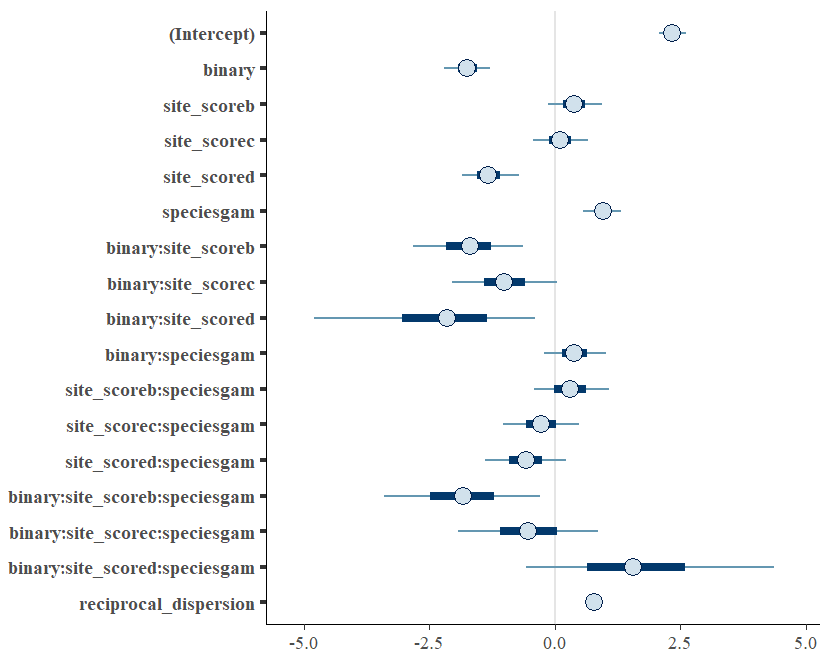 | | | | | |

**Table B:** Summary of the statistical models fitted to the Côte d’Ivoire entomological data. In each case, a Bayesian framework is applied that includes 4 chains with 1,000 iterations burn-in. In all models, notation $x_{1}$ is an indicator for pre (0) or post (1) intervention described by coefficient $\beta_{1}$; $x_{2}$ is an indicator for control (0) or treatment (1) arms ($\beta_{2}$). Diagnostics shown include: i) Monte Carlo standard error (mcse) – confirming this is low relative to the posterior standard deviation otherwise uncertainty could be masked; ii) The effective posterior sample size (ESS), given MCMC draws are not independent, may show some autocorrelation. The information about the posterior are more independent when the ESS is lower and the approximate number of draws with equivalent estimated accuracy is indicated by the *n_eff_* – values above 1,000 is more than sufficient; iii) the potential scale reductor factor (Rˆ) to check within and between chain variance is similar, convergence returns a value close to 1; iv) visual checks to confirm chain convergence, following (1). Adjusted scale for priors are noted.

| Model 1: Côte d’Ivoire | $\theta_{i}=e^{\beta_{0}+\beta_{1}x_{1i}+{\beta_{2}x}_{2i}+ \beta_{4}x_{1i}x_{2i}+(1\vert village)}$  $Y_{i}\sim NB(\mu_{i}\theta_{i}, \varphi)$  Normally distributed priors, *N ~* (*0, scale*) | | | | |
| --- | --- | --- | --- | --- | --- |
| Diagnostics: | mcse | *n_eff_* | Rˆ | Adj-scale | Visual confirmation of chain conversion |
| Intercept ($\beta_{0}$) | 0.0 | 1924 | 1.0 | | 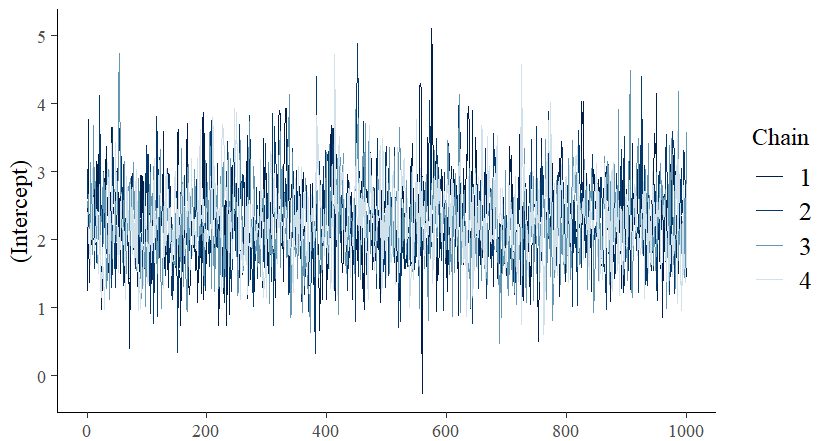 |
| $\beta_{1}$ | 0.0 | 2176 | 1.0 | 12.74 | 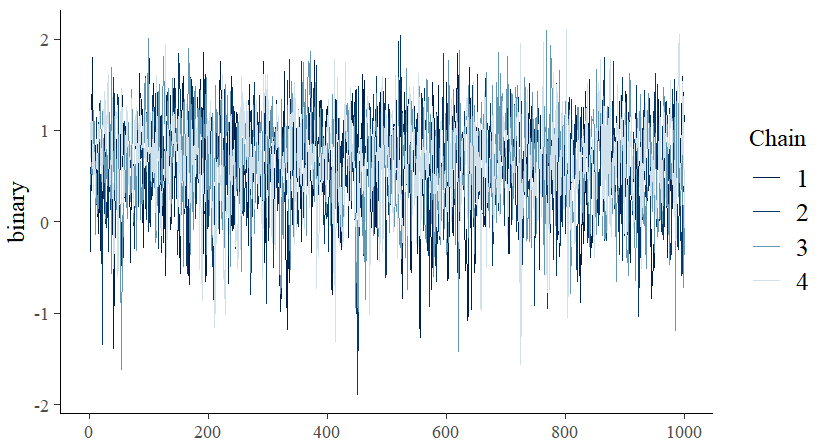 |
| $\beta_{2}$ | 0.0 | 1640 | 1.0 | 5.00 | 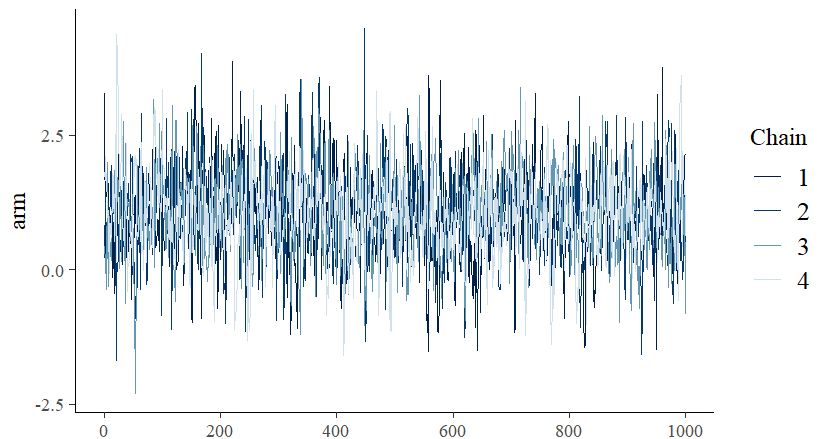 |
| $\beta_{4}$ | 0.0 | 1915 | 1.0 | 5.00 | 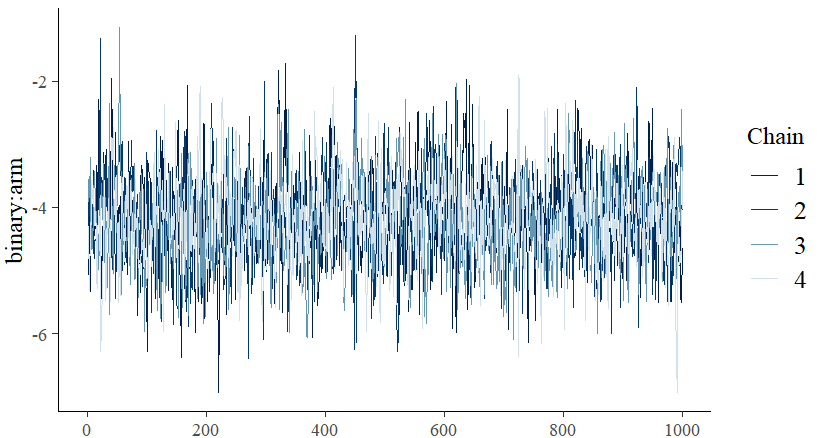 |
| b[(intercept) Kolékaha]  b[(intercept) Lofinékaha]  b[(intercept) Kakologo]  b[(intercept) Nambatiourkaha] | 0.0  0.0  0.0  0.0 | 2206  2281  2165  2289 | 1.0  1.0  1.0  1.0 | | 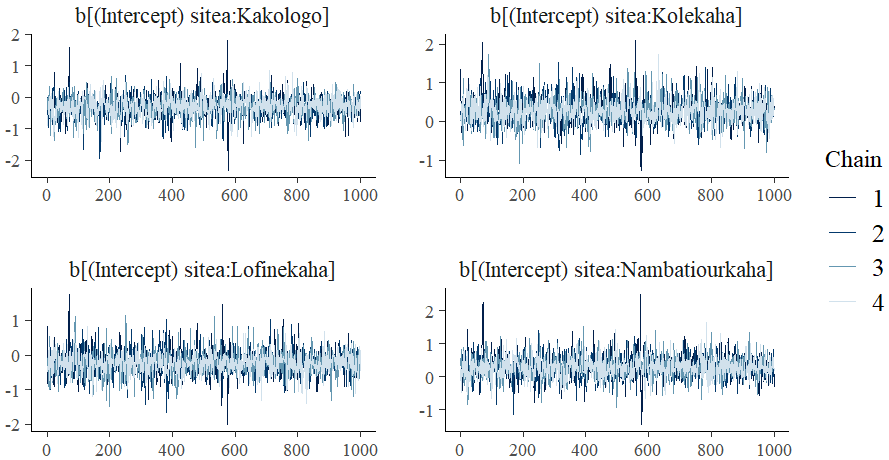 |
| Reciprocal dispersion  Sigma[Kolékaha:(intercept), (intercept)] | 0.0  0.0 | 3715  1970 | 1.0  1.0 | | 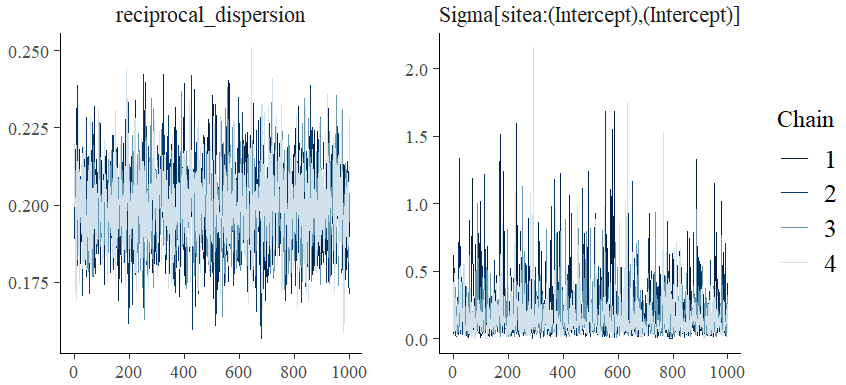 |
| 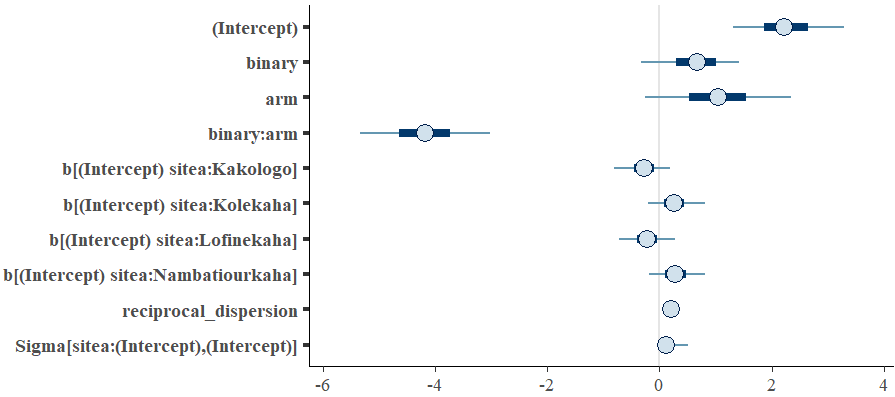 | | | | | |
| Model 1: Côte d’Ivoire | $\theta_{i}=e^{\beta_{0}+\beta_{1}x_{1i}+{\beta_{2}x}_{2i}+ \beta_{4}x_{1i}x_{2i}+(1\vert village)}$  $Y_{i}\sim NB(\mu_{i}\theta_{i}, \varphi)$  Normally distributed priors, *N ~* (*0, scale*) | | | | |
| Diagnostics: | mcse | *n_eff_* | Rˆ | Adj-scale | Visual confirmation of chain conversion |
| Intercept | 0.0 | 1029 | 1.0 | | 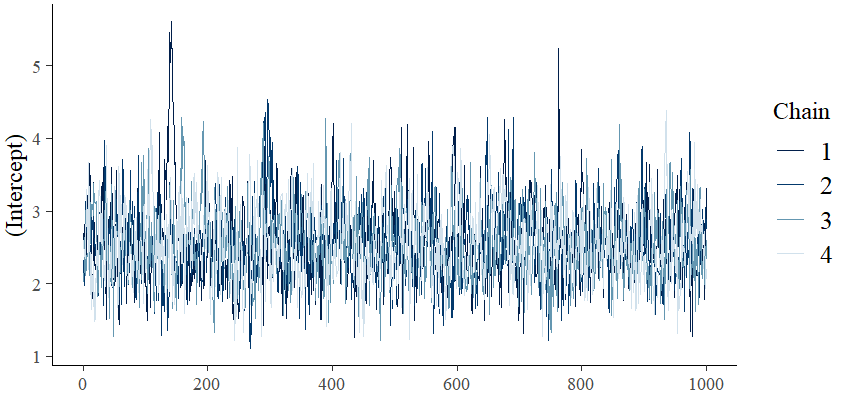 |
| $\beta_{1}$ | 0.0 | 1044 | 1.0 | 12.74 | 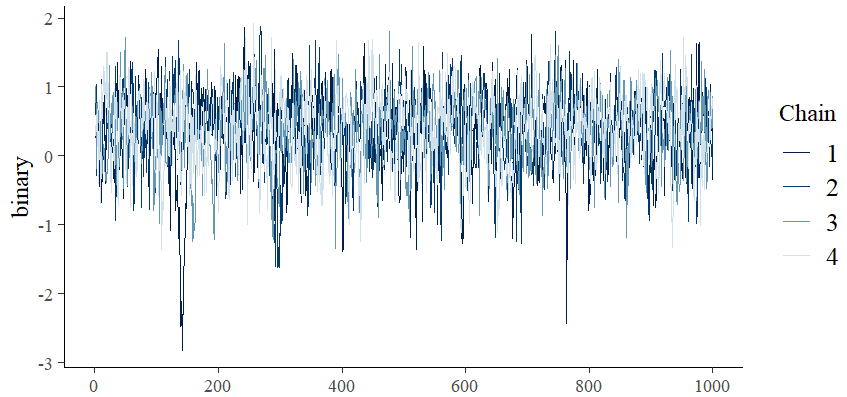 |
| $\beta_{2}$: Kakologo  $\beta_{2}$: Nambatiourkaha | 0.0  0.0 | 1115  1164 | 1.0  1.0 | 5.52  5.86 | 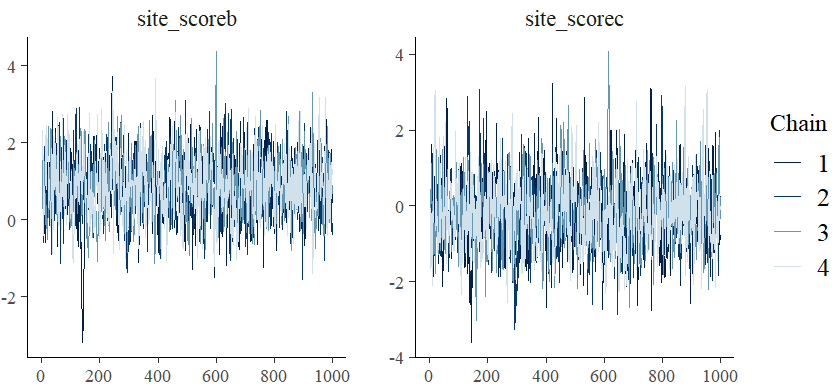 |
| $\beta_{4}$: Kakologo  $\beta_{4}$: Nambatiourkaha | 0.0  0.0 | 1130  1214 | 1.0  1.0 | 5.60  5.94 | 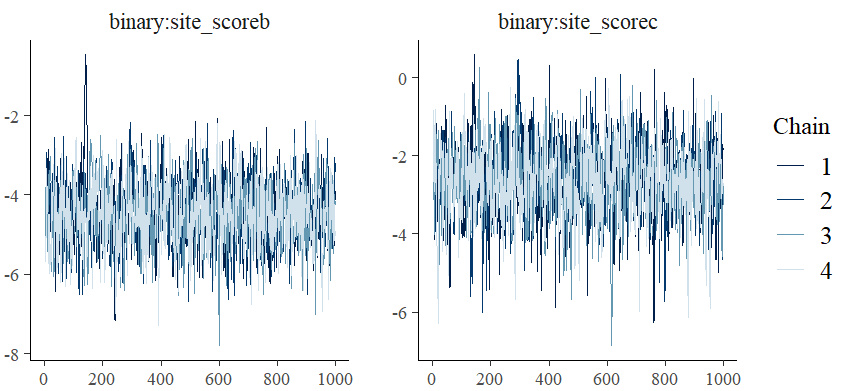 |
| Reciprocal dispersion | 0.0 | 3277 | 1.0 | | 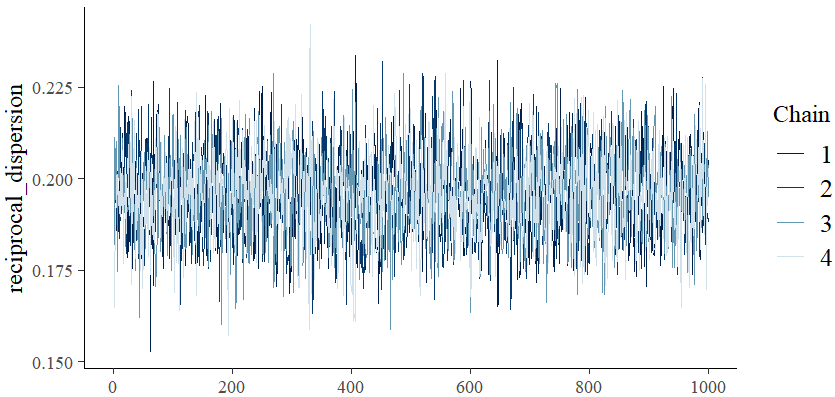 |
| 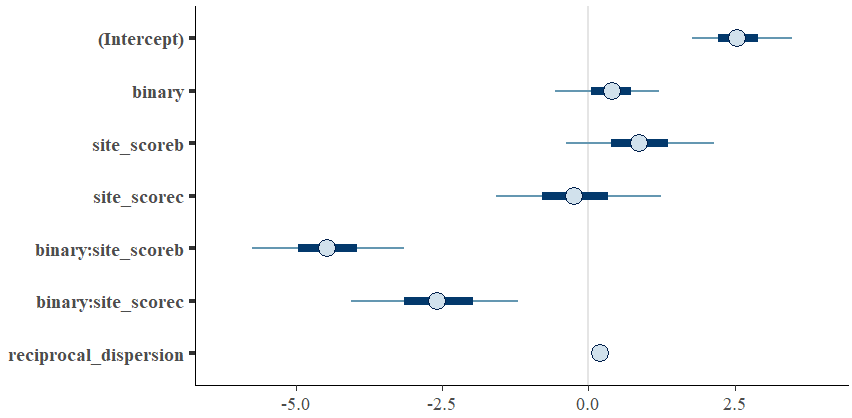 | | | | | |

**Table C**: Summary outputs from transmission model simulations using reductions in larval mosquito density as estimated empirically at the village level in Côte d’Ivoire, uncertainty in parentheses show 90% uncertainty interval.

|  | Kakologo | Nambatiourkaha |
| --- | --- | --- |
| Larval density estimated relative reduction (%) | 98.9% (96.1% – 99.7%) | 92.6% (69.0% – 98.4%) |
| Absolute reduction in vectors per person | gambiae: 51.3 (46.9 – 53.3)  other: 7.0 (6.6 – 7.3) | gambiae: 7.3 (5.0 – 8.0)  funestus: 1.0 (0.7 – 1.1)  other: 0.16 (-0.05 – 0.22) |
| Model estimated relative reduction in all-age prevalence (%) | 51.9% (44.3% – 58.8%) | 30.5% (24.3% – 62.5%) |
| Absolute fewer clinical cases in follow-up year compared to baseline | 807 (720 – 842) fewer cases per 1,000 people | 166 (242 – 546) fewer cases per 1,000 people |
| Relative reduction in all-age clinical incidence (%) | 94.7% (84.5% – 98.7%) | 83.4% (44.2% – 99.7%) |


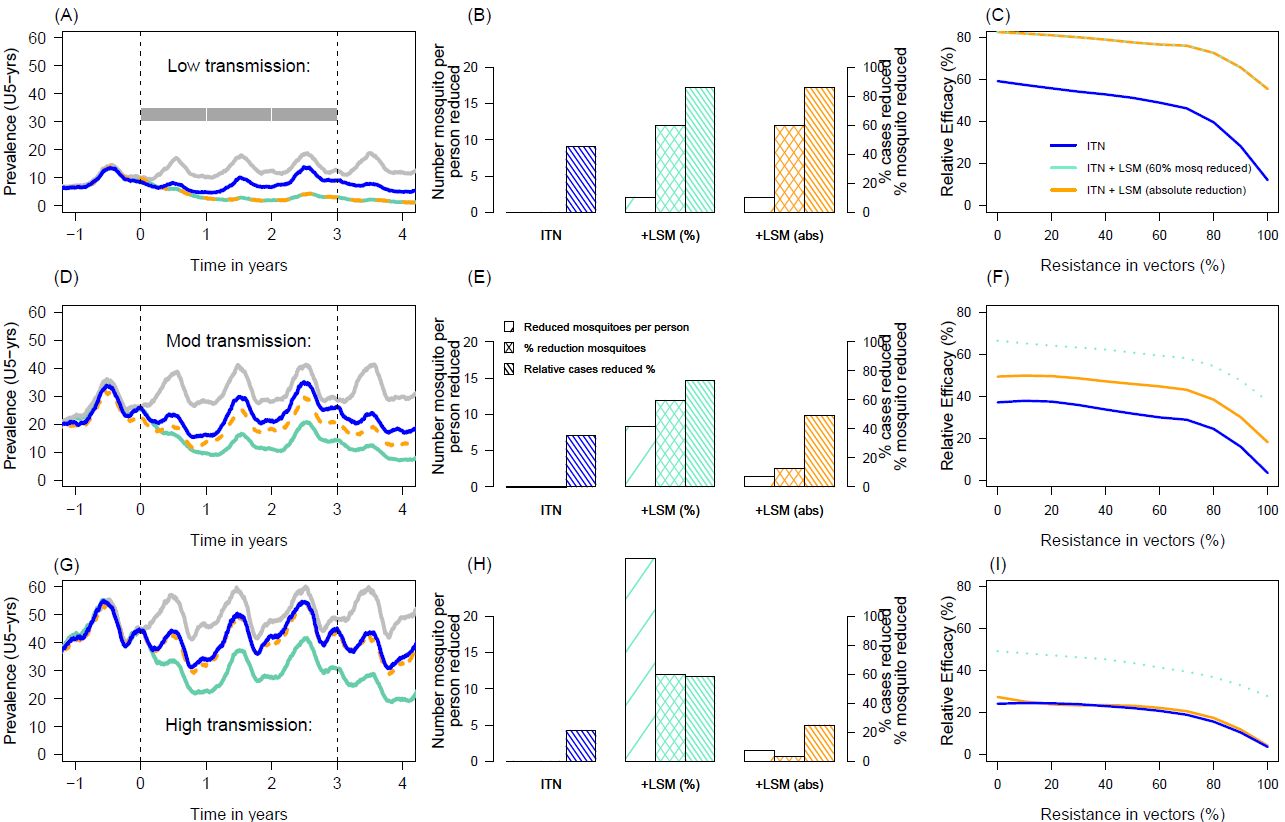


**Fig A. Theoretical impact of larval source strategies that suppress *Anopheles* mosquitoes.** Panels A, D and G show the prevalence in children of 6 to 59 months of age as measured by microscopy over time when endemic malaria burden is low, moderate or high respectively. In these scenarios, 80% of local mosquitoes are assumed to be resistant to pyrethroid insecticide. Simulations are parameterized so that 87.2% of mosquito foraging attempts for blood meals take place in bed. The impact from ITNs used by 60% of the population with waning impact over time (blue line) is compared to 60% ITN use plus larviciding efforts that either: i) suppress mosquitoes densities by 60% (green line) from time 0 onward, or; ii) reduce the absolute number of mosquitoes by the same number as a 60% reduction in the low transmission setting. Outcomes are compared to a counterfactual simulation (grey line) were no new interventions are deployed from time 0. Panels B, E and H: The absolute number of mosquitoes reduced per person in the transmission model scenario, the corresponding relative reduction in vectors, and the relative all-age cases averted over 3 years are shown for the simulations deploying ITNs only (blue), those deploying ITNs and LSM where 60% reductions in vector densities are simulated (green), or the removal of the absolute number of mosquitoes that aligns with the low transmission scenario (thus, middle green and orange bars match in panel 1B). Panels C, F and I: Relative efficacy over 3 years [relative reduction in parasite prevalence in children under 5 years of age compared to no intervention] when deploying ITNs (blue), ITNs with LSM that reduces 60% of mosquitoes (green), or ITNs with LSM reducing the same number of mosquitoes as would achieve a 60% reduction in the low transmission scenario (orange) versus different levels of pyrethroid resistance in local mosquito populations.


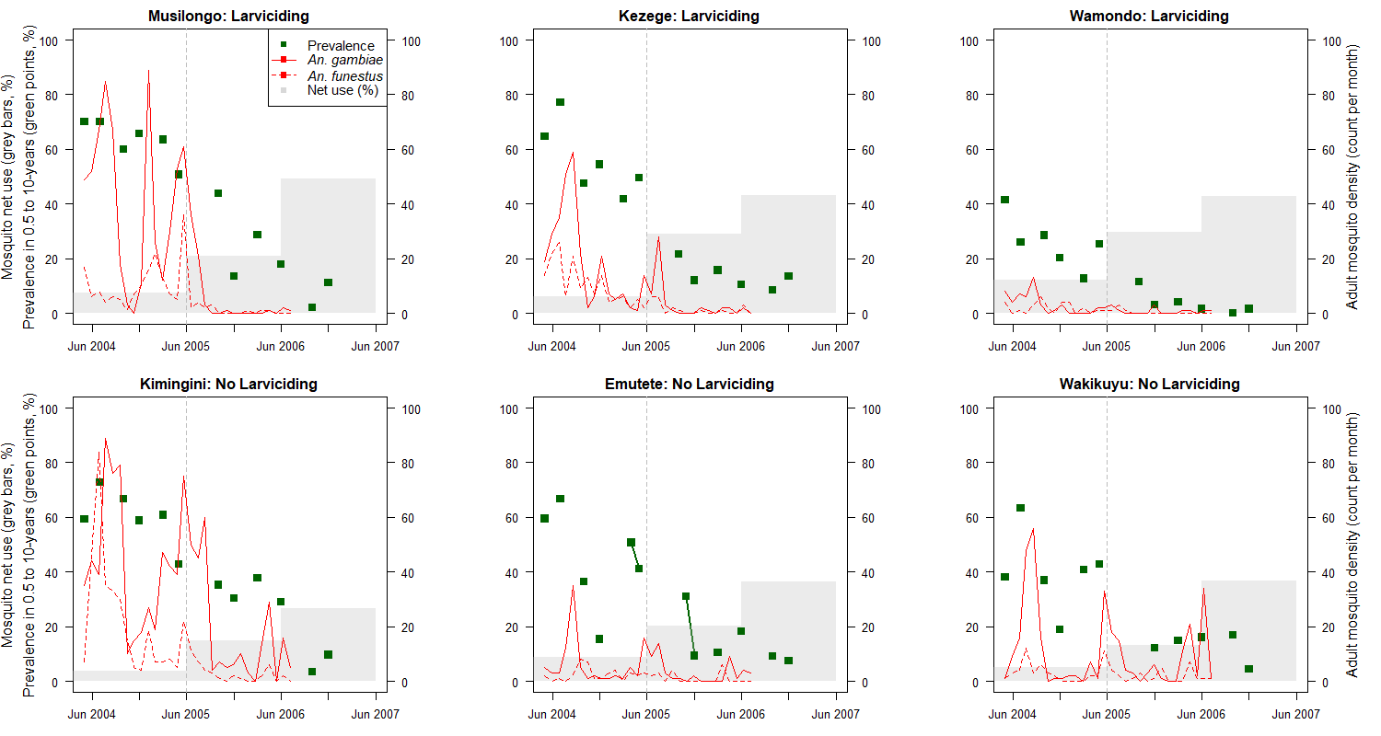


**Fig B. The observed data for six sentinel villages tracked during a larviciding randomized control trial in Kenya** (2). Each panel shows a sentinel village. ITNs were continually increased in all villages, education on mitigation increased across villages and larviciding with *Bti* bio-larvicide was applied weekly in larviciding locations (upper panels). The estimated relative densities of adult *Anopheles* mosquitoes by species complex are shown in red (*An. gambiae* s.l. solid lines; *An. funestus* s.l. dashed lines). The prevalence in children 6-months to 10-years of age are depicted by green squares. The average net use among the community each year after the onset of the trials is shown in grey bars (see Figure S3 for ITN use as simulated in the modelling exercise). The dashed vertical line indicates the onset of the larviciding application in June/July 2005.


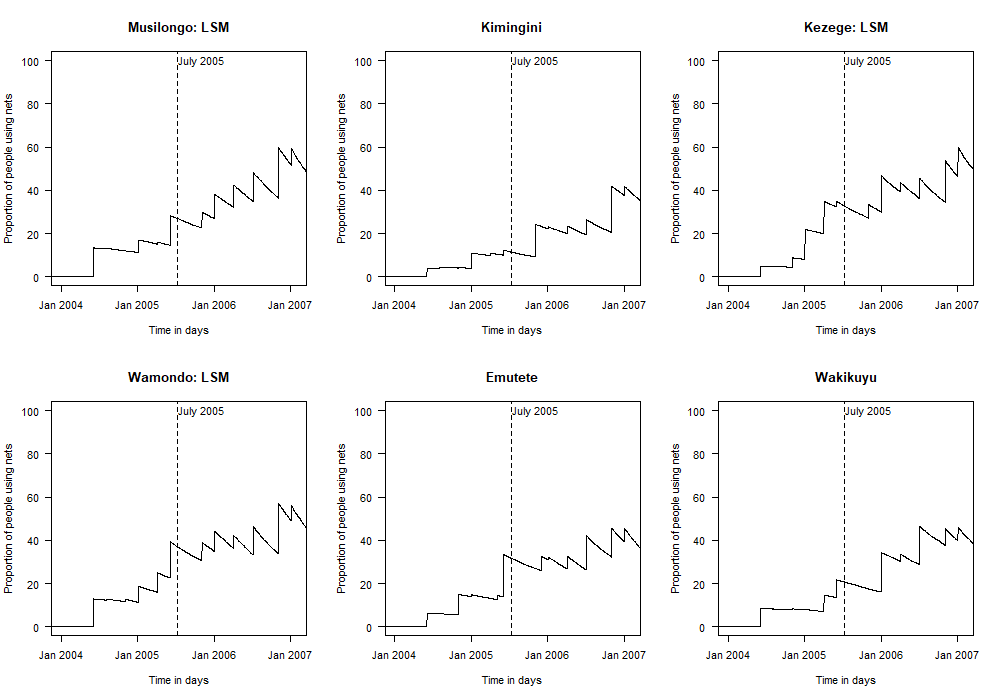


**Fig C.** Net use measured in the tracked cohort of children of 6-months to 10-years of age at cross sectional surveys throughout the Kenyan trial. Estimated using the proportion of children reporting to have slept beneath a net the previous night when asked during the survey. The villages of Musilongo, Kezege and Wamondo also received weekly *Bacillus-*larviciding treatments of local breeding sites from June 2005 for 19 months. All sites progressively increased LLIN use throughout the study.


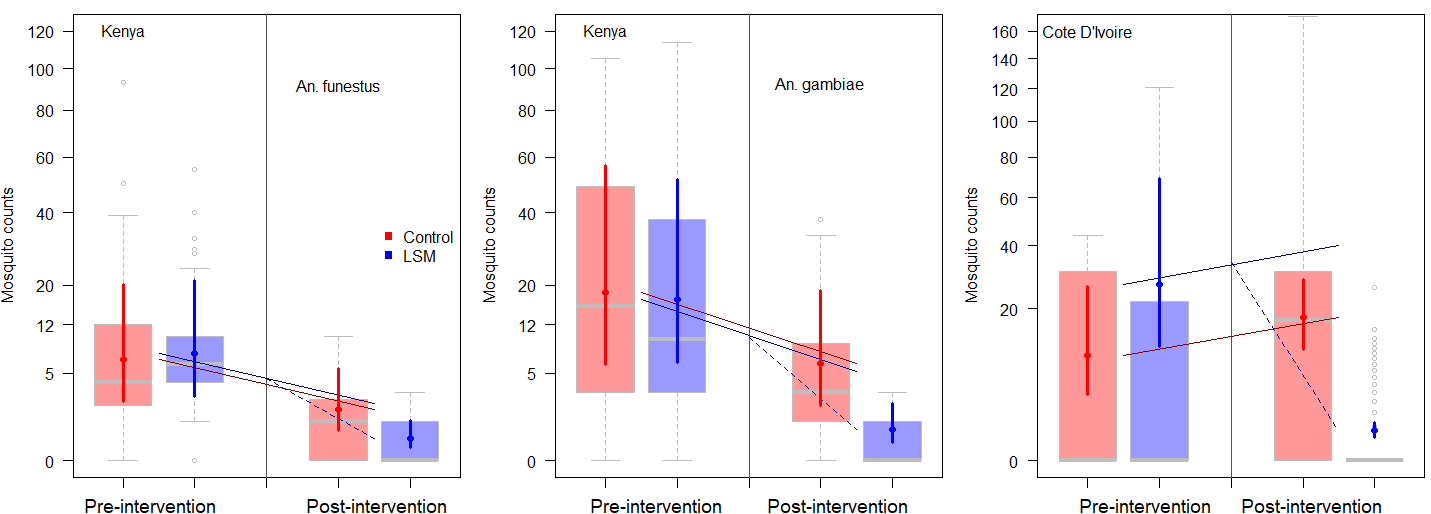


**Fig D. Summary results for the crude difference-in-difference estimates**. Kenya data split into panels A and B, for *An. funestus* and *An. gambiae* independently. Côte d’Ivoire results in panel C. In all panels, boxplots show the empirical data mosquito count ranges for, in order: the combined control arm counts prior to intervention, the corresponding larviciding trial arm, the post-intervention control data, and the corresponding larviciding arm post-intervention data. The statistical estimates from the difference-in-differences analysis to confirm model fit (vertical lines show 90% credible intervals with point estimate for median). The straight red lines indicate the change from pre to post intervention in the control villages, the solid blue line indicates the same amount of change adjusted for the pre-intervention mosquito counts. The dashed blue line indicates the additional benefit from the LSM.

**
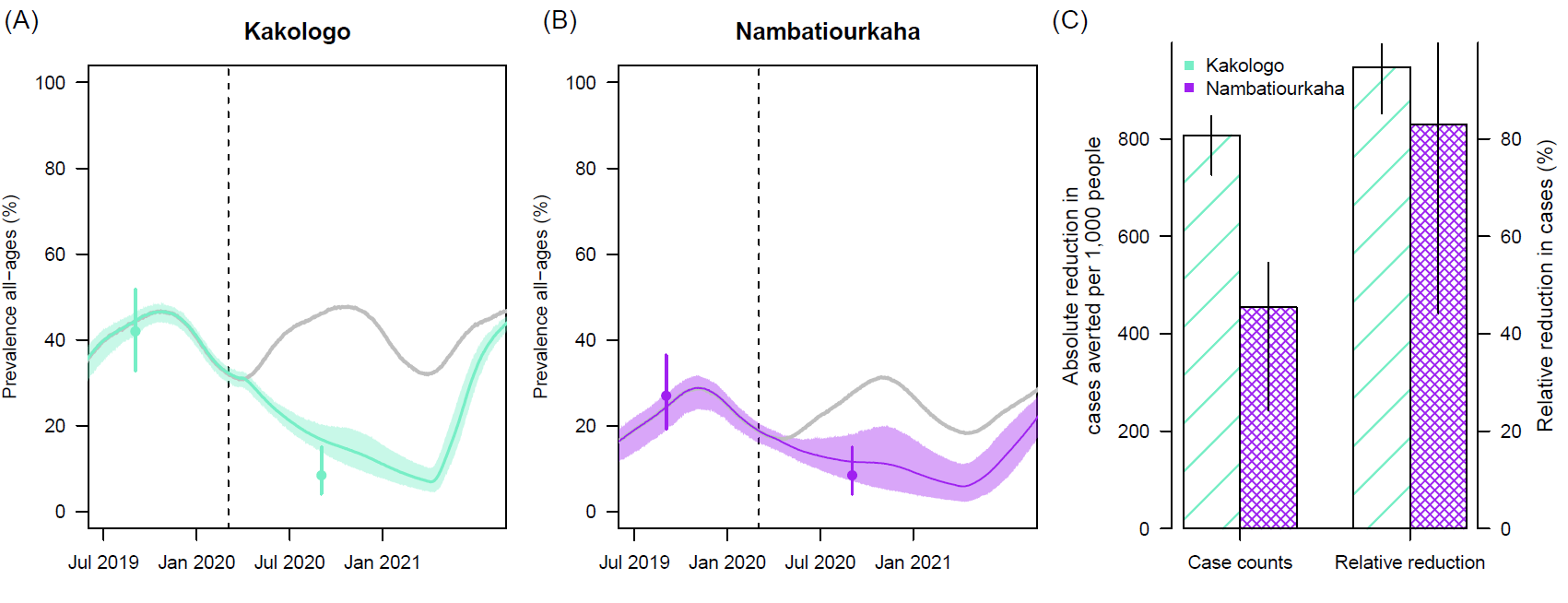
**

**Fig E.** **Larval densities analysis and model simulated results for the Côte d’Ivoire trial.** (A-B) Model simulated estimates of all-age prevalence in the villages of Côte d’Ivoire given the larval density data is used to parameterise the larviciding impact. In treatment villages (A: Kakologo, and B: Nambatiourkaha), grey lines indicate the counterfactural where parameters are matched but no effect from larvicide is simulated. Estimated prevalence from trial information are shown but were never empirically measured, included here to serve as an indicator of the capacity of the model to broadly reproduce the scale of impact observed using the health facility data from the trial (3). Uncertainty intervals from model parameter sensitivity analysis shown in A-B as polygons around mean estimated prevalence trend. C) Absolute (left axis), and relative (right axis) estimates of the reduction in clinical cases in the follow-up year in comparison to the baseline year for each treatment village Kakologo (light green), and Nambatiourkaha (purple).

**References**

1. Muth C, Oravecz Z, Gabry J. User-friendly Bayesian regression modeling: A tutorial with rstanarm and shinystan. Quant Method Psychol. 2018 Apr 1;14(2):99–119.

2. Fillinger U, Ndenga B, Githeko A, Lindsay SW. Integrated malaria vector control with microbial larvicides and insecticide-treated nets in western Kenya: a controlled trial. Bull World Health Organ [Internet]. 2009 Sep [cited 2018 Mar 7];87(9):655–65. Available from: http://www.ncbi.nlm.nih.gov/pubmed/19784445

3. Tia JPB, Tchicaya ESF, Zahouli JZB, Ouattara AF, Vavassori L, Assamoi JB, et al. Combined use of long-lasting insecticidal nets and Bacillus thuringiensis israelensis larviciding, a promising integrated approach against malaria transmission in northern Côte d’Ivoire. Malar J. 2024 Dec 1;23(1):168.
