# Supplementary material for: Heterogeneous impacts for malaria control from larviciding across villages and considerations for monitoring and evaluation
Source: PLoS Pathog. 2025 Jul 28;21(7):e1013287. doi: 10.1371/journal.ppat.1013287 (PMC12321148; doi:10.1371/journal.ppat.1013287)
Supplement: Attachment — Submitted filename: Response to Reviewers.docx [file ppat.1013287.s005.docx]

Dear Editor,

Thank you for the positive and constructive reviews and opportunity to resubmit our article: “**Heterogeneous impacts for malaria control from larviciding across villages and considerations for monitoring and evaluation**”. We have – point-by-point – answered the reviewer comments below. Here, line numbers relate to the cleaned manuscript. We would like to thank the reviewers for the time taken to constructively criticise and thus improve our contribution.

Please find attached in addition to this rebuttal letter:

* A marked-up copy of our manuscript that highlights changes made to the original version: '*Revised Manuscript with Track Changes'*.
* An unmarked version of our revised paper without tracked changes: '*Manuscript'*.

Sincerely, and on behalf of all authors,

Ellie Sherrard-Smith

**Reviewers' Comments:**

Reviewer's Responses to Questions

**Part I - Summary**
Please use this section to discuss strengths/weaknesses of study, novelty/significance, general execution and scholarship.

Reviewer #1: Very interesting and well-written manuscript on the impact of larviciding (as a supplementary tool, on top of bednets) on both mosquito abundance and malaria prevalence. It is unfortunate that the model did not (always) capture the observed trial data from Kenya and Cote d’Ivoire, but the authors rightly state that all villages receiving larviciding benefited from the intervention, and have done a wonderful job describing why modeling the impact of LSM is challenging. Their suggestions on how to improve entomological surveillance and measure other indicators are helpful.

Reviewer #2: The manuscript is providing indirect method or model for theoretical epidemiological benefits/impacts related to introduction of larval source management to supplement the core vector control intervention, the ITNs for malaria control and achieving its elimination. The study is cost effective as it can rely only on entomological parameters such as density of adult malaria vectors to predict the impact on averted malaria cases.

Reviewer #3: The authors present a compelling analysis of larval source management (LSM) as a complementary intervention for malaria control, using an individual-based transmission model parameterized with empirical data from two well-characterized trials in Western Kenya and Côte d’Ivoire. The study is methodologically strong, timely, and relevant for both implementation science and vector control policy.
The manuscript makes important contributions by showing that empirically measured reductions in adult mosquito densities—despite site-level heterogeneity—can be used to parameterize dynamic transmission models and broadly reproduce observed epidemiological outcomes. This is particularly valuable in a field where direct epidemiological evidence for LSM remains limited.
The manuscript is generally well-written, thorough, and scientifically sound. That said, I believe the following revisions and clarifications would strengthen the work and increase its value to the broader research community.

**Part II – Major Issues: Key Experiments Required for Acceptance**
Please use this section to detail the key new experiments or modifications of existing experiments that should be absolutely required to validate study conclusions.

Generally, there should be no more than 3 such required experiments or major modifications for a "Major Revision" recommendation. If more than 3 experiments are necessary to validate the study conclusions, then you are encouraged to recommend "Reject".

**Reviewer #1**: It is not clear to me why only one LSM strategy (that reduces the number of female Anopheles by 60%) is used. What is your rationale? What would the outcome be if its impact is higher or lower? I also do not understand why then a reduction in the absolute number of mosquitoes by the same number as a 60% reduction in the low transmission setting is used. I could not find the reasoning for that.

*RESPONSE: The reviewer raises a valid point that it could be useful to also consider other % reductions than 60%. The major take home remains that the impact from an absolute reduction of mosquitoes can be quite different between transmission settings. Our choice of the 60% reduction is arbitrary but the aim of the first section was to demonstrate that the effort needed is quite distinct in different settings and that if there are large habitat sections to address for successful larval management, then we need larger teams to have a particular impact (% reduction) relative to settings with fewer more accessible breeding sites. The model is used to show that a given percentage reduction in vectors (60%) in the low transmission setting corresponds to a given absolute number of mosquitoes. If we were able to reduce that number of vectors from a different site, if transmission is higher, then the impact would be less.*

*We have added at lines 212 – 214:* “(The value of the percentage reduction achieved in the low transmission setting is arbitrarily selected for illustrative purposes but allows us to estimate absolute vectors per person from the model.)”

I would ask the authors to copy some critical information (which is now in the methods section) to the results section, so the readers understand the context early on. For example, can you add the type of bednet, period(s) and frequency of larviciding (weekly Kenya, every fortnight Cote d’Ivoire) before showing the relative reduction in adult mosquito densities?

*We have added: Lines 311 – 317: “*Standard pyrethroid-treated nets (ITNs, PermaNet 2.0 and Olyset Nets) were gradually introduced in all communities, in addition to the intervention in the trial. The larviciding and non-larviciding arms of the trial were selected so that the baseline number of aquatic habitats were roughly equal in communities in each arm. Larval and adult mosquito densities were monitored during the year prior to larviciding and throughout the trial. The larviciding communities received weekly larviciding treatments to all accessible habitats.*”*

*And adjusted lines 406 - to read: “*Data from Côte d'Ivoire came from 4 villages (2 were treated with LSM and 2 were monitored as comparisons) in the department of Napiélédougou in Korhogo Health District where communities had received pyrethroid-only ITNs (PermaNet 2.0) via the mass campaign that took place in 2017 (35). Larviciding was conducted fortnightly outside the times of heavy rain with larviciding treatment events occurring across the follow-up year (35).”

Discussion: You may want to add the (likely) increased relative impact of proper LSM strategies (on top of bednets) in areas with resistant mosquitoes and/or areas where mosquito behaviors reduce contact with ITNs/IRS.

*Thank you for the suggestion, we agree that there will be a likely benefit of properly delivered LSM strategies in areas with resistant vectors, but we do not have the data to investigate this explicitly here.*

*Lines 524 – 526: “*Similarly, activity of humans and vectors (rendering vector contact with ITNs less frequent or leaving people more exposed) that drive the degree of residual transmission will alter the potential impact from larvicide and ITNs (15).*”*

Discussion: Throughout the text there is a wealth of information on confounding factors and suggestions on how to improve surveillance. I would ask the authors to create a box, summarizing the critical indicators that should (ideally) be measured to improve modeling exercises, and how those may differ between low/medium/high transmission settings.

*We considered the Box 1 suggestion, however we could not find any guidance for including this within the journal guidelines nor an online example of other manuscripts from this publication including such a Box. We are open to the suggestion but request editorial guidance on whether it is acceptable. Thank you for the suggestion and we hope that at least we have included critical points within the discussion.*

**Reviewer #2**: I have only minor issues which will be addressed by the authors of the manuscript.

**Reviewer #3**: Major Comments
1. Clarify the rationale for using adult mosquito density over larval metrics While the discussion acknowledges practical limitations of using larval data, the manuscript could better articulate why adult densities—rather than larval or pupal indices—were chosen as the principal metric for model parameterization. Given that larval productivity varies and not all larvae translate into adults, readers would benefit from a clearer explanation of the trade-offs involved in this decision, especially for settings with complex aquatic habitats.

*RESPONSE:.*

*In the first submission, we noted that “*The [Côte d'Ivoire] trial noted that all larval sites that were findable were also treated. This indicates that any unfound larval habitat contributing to adult females in the region would not contribute to relative reduction in larval density estimates, thus potentially over-estimating impact.”

*In some locations it is challenging to source or reach habitats (although this is being addressed somewhat with technology advances). The adult is the critical vector life-stage for transmission events so that it is important to track the effects of control strategies on the relative density of this life stage. By focusing on the adult, we are avoiding some of the challenges in the complex dynamics of earlier life stages.*

*We have included:*

*Lines 600 – “*The larval reductions were impressive in Côte d’Ivoire, using reductions determined from the larval data within the transmission model, greater impacts were estimated than the empirical data observed (Figure S5, Table S3). The trial noted that all larval sites that were findable were also treated. This indicates that any unfound larval habitat contributing to adult females in the region would not contribute to relative reduction in larval density estimates, thus potentially over-estimating impact. Another potentially critical metric is the proportion of larval habitats that are found that contain pupae (or late stage larvae). Not all larvae will successfully pupate to adult stage vectors, so even where larval densities are high, the emergence of adults may be hard to predict. A limitation of our theoretical modelling exercise is that we do not consider the variability in productivity between larval habitats, nor stages of larvae present. The exercise was used to show how the same effort in two places might achieve different relative reductions, however, it might be easier to find highly productive larval sites and managing these through LSM would then have greater benefit (would reduce the overall densities by a greater degree). Similarly, larval sites in a high transmission location may be generally more productive and rapidly found, so the absolute number of mosquitoes killed may be far higher than for low transmission locations per unit effort.*”*

*And Lines 631 – “*An important consideration is appropriately powered entomological sampling and the methods used to count mosquitoes. Sampling mosquitoes is notoriously challenging (64–67) and many studies are considered under-powered to detect changes in densities, sporozoite rates, foraging behaviours and other critical aspects of mosquito ecology that are associated with malaria transmission. Very clear reductions in adult densities, however, were measurable in the Kenyan and Côte d’Ivoire trials given the committed follow up effort from both trial teams. In Kenya, the trial authors noted that the use of pyrethrum spray catches for adult vectors may have underestimated absolute densities given the opportunity for mosquitoes to have left the house prior to catch teams operating in the morning (44). This would be however a consistent concern across villages. Evidence suggests the method used to record adult densities and species compositions can return variable results, with sampling methods around housing tending to target anthropophilic species and sampling elsewhere perhaps better representing opportunistic species (68–70). Therefore, the entomological method to employ to assess LSM impacts must consider these challenges, perhaps combining different approaches as was done in Côte d’Ivoire where by pyrethrum spray catches and window traps were used (35), though this particular method combination may still draw the focus onto house-dwelling species. Developing entomological methods that are highly powered and precise will prove key for monitoring environmental interventions. An alternative indicator that could be used is the proportion of habitats that contain late stage malaria vectors (pupae) at any given time, as a regular application, should prevent that and consequently lead to a reduction in adult vectors. Future work could consider how this metric might be predictive of epidemiological impacts.*”*

2. Expand discussion on model validation in Côte d’Ivoire. The Côte d’Ivoire trial relied on retrospective health facility data with substantial village-level variability. While the authors attempt to calibrate model outputs to estimated prevalence, further discussion on the robustness of these estimates—especially considering health-seeking behavior, facility reporting rates, and catchment uncertainties—would be valuable. Are alternative validation strategies or uncertainty propagation approaches feasible?

*This is a valid point. We have added at lines 589 – 599: “*The Côte d’Ivoire trial relied on retrospective health facility data with substantial village-level variability. In this modelling exercise, we tried to infer from these clinical incidence data on prevalence trends and impacts. There are well-documented challenges with using health facility data for trends analyses: treatment-seeking behaviour can differ between and among communities (59,60), there is uncertainty in the population sizes served by some health facilities which can complicate trend interpretation (7), there are different approaches to treating and counting patients presenting with fevers as malaria cases (60,61) and potential shortages or stock-outs in equipment that impact surveillance efforts (60–62). These uncertainties represent a limitation for our analysis. As an alternative method antenatal care trends could be used (61) but were not available for our analysis.”

3. Strengthen the link between entomological reduction and transmission heterogeneity. The model performs well in explaining broad trends, but the linkage between mosquito density reductions and heterogeneous epidemiological outcomes could be expanded. For instance, do variations in species composition, net use, or behavioral plasticity in biting patterns explain the differing impacts across villages? Figure 5F suggests some signal, but the interpretation could be more clearly developed in the discussion.

*These are useful inferences. In the model, species composition and biting patterns are somewhat linked because the proportion of biting indoors or in bed, and propensity to bite humans for the blood meal are species specific assumptions. We have tried to suggest how empirical data and understanding of context may improve modelled scenario outcomes. We have also added to the discussion, as per lines 514 – 534:*

*“*Reductions in adult mosquito densities can be attributed to larvicide applications and deliver locally specific epidemiological outcomes. While it is key to note that all villages receiving larviciding benefited from the intervention, there was not a clear association between the estimated absolute number of mosquitoes reduced and the ultimate epidemiological outcome in the different villages in Kenya and Côte d’Ivoire. Although, there appears to be some linkage between mosquito density reductions and epidemiological outcomes (Figure 5), with higher burden settings showing generally higher absolute reductions in vectors using model derived estimates. There are several potential reasons for the differences that we observe. First, there were differences between villages in the use of ITNs, particularly in Kenya (34), that could complementarily contribute to transmission reductions. Similarly, activity of humans and vectors (rendering vector contact with ITNs less frequent or leaving people more exposed) that drive the degree of residual transmission will alter the potential impact from larvicide and ITNs (15). Differences in the microclimate may exist that alter key elements such as the gonotrophic cycle (46,47), extrinsic incubation period (48) or other life traits of vectors (49). The time from egg laying to pupation is variable with estimates having been reported to vary between 5 and 14 days (50), the trials applied larvicide either weekly (44) or fortnightly (35) so that if micro conditions differed between villages, which could enabled different rates of passage through the aquatic life stages, adult mosquito emergence rates could also feasibly differ. In Côte d’Ivoire this may be a contributing explanation for the lower impact of larviciding efforts against adult densities in Nambatiourkaha, in contrast to Kakologo (Table 2).*”*

4. Discuss spatial dynamics and vector movement The assumption of spatial isolation is acknowledged, but the model does not simulate spillover effects or vector migration across untreated areas. While this is understandable for tractability, a short discussion on the implications of this omission—particularly for scalability or real-world program design—would add nuance.

*We have added: Lines 690 – 700: “*We also do not simulate spillover effects or vector migration across untreated areas in the modelling exercise. Spatial modelling approaches (75) may be useful to consider how *Anopheles* dispersal, approach to aestivation, and recolonization following disturbance across communities could drive further heterogeneity in LSM impacts although little data currently exists on the transience of such ecological conditions. We would expect such challenges to affect LSM outcomes depending on how lasting implemented changes prove to be in real-world contexts, or how neighbouring locations are managing malaria vectors. Another limitation of the modelling is that we assume reducing mosquito densities does not alter the vector competence of the different species. It is still unclear how density dependent larval development impacts vector competence in field settings, though results from the laboratory suggest it could be substantial (76–78).*”*

5. Comment on implications for non-larviciding forms of LSM The manuscript emphasizes larviciding, but the introduction and discussion frame LSM more broadly (including habitat manipulation and removal). A short comment on whether these findings are likely to translate to non-chemical approaches would increase the manuscript's applicability to integrated vector management (IVM).

*Thank you, we recognise that the studies we examined applied a larvicide (Bti) so that we focused on this as the intervention. That said, it was our intention to use a mechanism in the transmission model that could represent any form of intervention that has capacity to reduce and maintain reductions of adult mosquitoes. The code removes a need to capture density dependent processes and simply alters the carrying capacity. We have added: Lines 708 – 713: “*One advantage of the modelling approach we have taken is that it is agnostic of the LSM strategy deployed as long as the impact achieves some reduction in mosquito densities. Further entomological, epidemiological and economic studies are needed in different settings to extend the evidence base to demonstrate how LSM can be best used as an effective malaria control tool.*”*

**Part III – Minor Issues: Editorial and Data Presentation Modifications**
Please use this section for editorial suggestions as well as relatively minor modifications of existing data that would enhance clarity.

**Reviewer #1**: L254 (figure 1)
Fig 3H: y-axis label partially overlaps with values

*Corrected, thank you*

L480 ‘unlike situation moving forward’ can you explain what you mean?
*We mean that untreated nets are not likely to be the core intervention for communities. Those with nets are most commonly using insecticide treated ones. To clarify, we have added, at lines 505 – 508: “*In a recent Cochrane review, another study from Tanzania had a similar format (44,45) but compared outcomes to untreated nets (36), but this is an unlikely situation moving forward as communities will be nearly universally using ITNs, so we chose not to include the study here.*”*

623-625 plus the residual efficacy of the product (?)

*Given some LSM are not insecticidal but more permanent changes we decided not to add this to the section, but thank you and we agree for commodities with waning duration this is a key component.*

**Reviewer #2**: I would appreciate the contribution of the authors by elucidating the added theoretical epidemiological benefit of larval source management to the ITNs, the primary core vector control intervention for malaria control by using indirect method, an individual-based transmission model for falciparum malaria. They used peered reviewed information from published data sets from Kenya and Cote d’Ivoire and presented the outcomes as well as the limitations that will guides future researchers.
Therefore, here below are minors concerns that require corrections or clarifications in order to improve the manuscript:

Abstract
In line 56, In Kenya, adult mosquitoes were sampled using Pyrethrum Spraying Catching (PSC) method and not CDC-light traps. Similarly, to mention the methods used for sampling adult mosquitoes in Cote d’Ivoire: Standard Window Traps and Pyrethrum Spraying Catching.

*Added*

Introduction
The introduction is well written and documented, but some below minor edits have to be considered.
- Line 76: To review the sentence as “the mass drug treatments is not a key intervention for malaria control. The referenced paper mentioned: " Prompt treatment of clinical malaria cases”.

*Thank you – corrected to: “*and the prompt treatment of clinical cases are key interventions used to control transmission*”*

- The last sentence between lines 79-81, requires a reference (see review copy highlighted in orange”.

*We have cited:*

World Health Organization. World Malaria Report 2024: addressing inequity in the global malaria response. Geneva, Switzerland: World Health Organization; 2024.

Selvaraj P, Wenger EA, Gerardin J. Seasonality and heterogeneity of malaria transmission determine success of interventions in high-endemic settings: a modeling study. BMC Infect Dis [Internet]. 2018 [cited 2018 Sep 12];18:413. Available from: https://doi.org/10.1186/s12879-018-3319-y

- The last sentence between 143-147 looks long and has to be spilt into two sentences. “first sentence ending by ----- conditions, and the second starting from ---- It is also hard….”

*Done*

- Line 155: At the sentence starting from line 155, the author used the word “suppression” of adult female mosquito density… The same word was used in other sections of the manuscript. This word seems not appropriate for malaria control strategy as most of core interventions aim the reduction of vector and malaria infection parameters. To find out an alternative word to replace “suppression”.

*We would prefer not to alter the text everywhere for this as we deliberately use the term suppression for LSM given the model mechanism we employ. In this case, the idea is to suppress the adult carrying capacity from its otherwise natural state. We agree with the reviewer that the ultimate aim is to reduce the transmission from vector to host and from host to vector transmission. We have changed this elsewhere when referring to the trial outcomes and empirical data.*

- Other edits are proposed on respective lines 163 and 168.

*Corrected*

Results: The results are supported by Figures and Tables. The following are the minor clarifications or changes:
- The types of ITNs distributed and evaluated in each country have to be included in the narrative text of results section (Standards PermaNet2 in Cote d’Ivoire; PermaNet and Standard Olyset Net in Kenya).

*We have added: Lines 311 – 317: “*Standard pyrethroid-treated nets (ITNs, PermaNet 2.0 and Olyset Nets) were gradually introduced in all communities, in addition to the intervention in the trial. The larviciding and non-larviciding arms of the trial were selected so that the baseline number of aquatic habitats were roughly equal in communities in each arm. Larval and adult mosquito densities were monitored during the year prior to larviciding and throughout the trial. The larviciding communities received weekly larviciding treatments to all accessible habitats.*”*

*And adjusted lines 406 - to read: “*Data from Côte d'Ivoire came from 4 villages (2 were treated with LSM and 2 were monitored as comparisons) in the department of Napiélédougou in Korhogo Health District where communities had received pyrethroid-only ITNs (PermaNet 2.0) via the mass campaign that took place in 2017 (35). Larviciding was conducted fortnightly outside the times of heavy rain with larviciding treatment events occurring across the follow-up year (35).”

- The annotations of figures (1,2,3,4,5) and tables (1,2). are mixing some narrative sentences which may be relocated in results or material&method sections. That means to shorten the annotations and keep only the naming of title, panels and other key figures of table and figures.

*We have made a decision to retain full legends as the journal editors allow because we intend these panels to be entirely interpretable. We have made some changes as suggested by the reviewer however – please see the tracked changes.*

- Figures 2 and 4 are missing annotations of cited panels.

*Corrected*

- The legend of Panel E, Figure 1 is not readable and needs the increase of the front size.

*Increased in size*

- The tables 1 and 2 require the improvement of formatting and improvement of presentations.
- Other minor corrections are proposed at the lines 334, 386.

*Thank you, we have edited, but following formatting instructions from the journal we have not altered the style as we understand the editorial team will advise on this if we are accepted for publication* [*Tables | PLOS Pathogens*](https://journals.plos.org/plospathogens/s/tables)

Discussion
This section is well written and documented. Some corrections/clarifications proposed at the lines 608 and 617.
Material & Methods and statistical analysis.
Some corrections are highlighted and proposed at the following lines: 674, 792, 804/805, 973 and 978.

*Thank you – corrections highlighted in the tracked changes version*

**Reviewer #3**: Minor Suggestions
* Figure 5: Panels are rich in information but could benefit from clearer legends or brief panel-specific descriptions in the caption, particularly for less familiar readers.

*Thank you – we have edited in line with the journal guidelines and these suggestions*

* Data accessibility: While code is well documented, the manuscript would benefit from a clearer summary in the main text of which datasets are fully publicly available and which are available upon request.

*Thank you, we shared all Kenyan data with the git repository. We have shared previously published data from Tia et al. 2024 here too. Tia Bleu will publish additional data from Côte d'Ivoire* *collected* *as part of their PhD and these data can be available on request. We have tried to make this more clear in the manuscript.*

Our Figures have been uploaded to PACE to ensure PLOS requirements are met.

We would like to thank all reviewers for timely and constructive critiques of our work. These reviews have improved the contribution.
